# Stress Granule-Mediated Oxidized RNA Decay in P-Body: Hypothetical Role of ADAR1, Tudor-SN, and STAU1

**DOI:** 10.3389/fmolb.2021.672988

**Published:** 2021-06-04

**Authors:** Ravi Kumar Alluri, Zhongwei Li, Keith R. McCrae

**Affiliations:** ^1^ Department of Cardiovascular and Metabolic Sciences, Lerner Research Institute, Cleveland Clinic, Cleveland, OH, United States; ^2^ Biomedical Science Department, College of Medicine, Florida Atlantic University, Boca Raton, FL, United States; ^3^ Taussig Cancer Institute, Cleveland Clinic, Cleveland, OH, United States

**Keywords:** oxidative stress, stress granules, P-bodies, oxidized RNA, 3′ UTR, ADAR1, Tudor-SN, STAU1

## Abstract

Reactive oxygen species (ROS) generated under oxidative stress (OS) cause oxidative damage to RNA. Recent studies have suggested a role for oxidized RNA in several human disorders. Under the conditions of oxidative stress, mRNAs released from polysome dissociation accumulate and initiate stress granule (SG) assembly. SGs are highly enriched in mRNAs, containing inverted repeat (IR) *Alus* in 3′ UTRs, AU-rich elements, and RNA-binding proteins. SGs and processing bodies (P-bodies) transiently interact through a docking mechanism to allow the exchange of RNA species. However, the types of RNA species exchanged, and the mechanisms and outcomes of exchange are still unknown. Specialized RNA-binding proteins, including adenosine deaminase acting on RNA (ADAR1-p150), with an affinity toward inverted repeat *Alus*, and Tudor staphylococcal nuclease (Tudor-SN) are specifically recruited to SGs under OS along with an RNA transport protein, Staufen1 (STAU1), but their precise biochemical roles in SGs and SG/P-body docking are uncertain. Here, we critically review relevant literature and propose a hypothetical mechanism for the processing and decay of oxidized-RNA in SGs/P-bodies, as well as the role of ADAR1-p150, Tudor-SN, and STAU1.

## Introduction

Cells constantly generate reactive oxygen species as byproducts of oxidative phosphorylation. The endogenous ROS generation can be regulated under many conditions (Droge, 2002; Martindale and Holbrook, 2002; Brand, 2010; Bae et al., 2011; Lee et al., 2011). In addition, exogenous oxidants may increase cellular ROS levels. The cellular antioxidant systems effectively reduce ROS and maintain a balance. Oxidative stress is a condition in which steady-state ROS levels are enhanced either transiently or chronically due to an imbalance of oxidants and antioxidants. While moderately elevated ROS under OS may lead to activation of cellular signaling pathways and disturbances in cellular metabolism, more severe or destructive stress causes the damage of cellular constituents and often cell death under acute or destructive stress conditions (Poli et al., 2004; Ryter et al., 2007; Lushchak, 2014). The extensive molecular and cellular damage caused by ROS may accumulate over time. Thus, OS is strongly implicated in many age-related and neurodegenerative disorders such as Parkinson’s disease, Alzheimer’s, atherosclerosis, and cancer, among others (Nunomura et al., 2001; Sayre et al., 2001; Hussain et al., 2003; Singh and Jialal, 2006; Vogiatzi et al., 2009; Nunomura and Perry 2020). Oxidative damage to DNA is a well-established consequence of oxidative stress and is strongly implicated in many diseases; however, less attention has been given to RNA oxidation. Previous studies in human lung epithelial cells revealed that in comparison with DNA, RNA has 14–25-fold more oxidative guanosine adducts when cells are challenged with hydrogen peroxide to induce OS (Hofer et al. (2005)), demonstrating the high sensitivity of RNA to oxidative damage. Moreover, mounting evidence has associated elevated levels of oxidized RNA with many age-related diseases (Zhang et al., 1999; Martinet et al., 2004; Chang et al., 2008; Kong et al., 2008). Some reports also indicate that RNA oxidation is an early event that precedes cell death (Shan et al., 2007), suggesting that cell lethality through this mechanism may directly promote human disease.

Excessive reactive oxygen species cause both physical and chemical damage to RNA, including strand breaks (Singh et al., 2004), induction of RNA cross-links (Jezowska-Bojczuk et al., 2002), and nucleoside base removal (abasic sites) (Tanaka et al., 2011), as well as numerous types of chemical base modifications. More than 20 oxidized base lesions have been identified in RNA secondary to the chemical action of ROS (Barciszewski et al., 1999) of which 8-hydroxyguanosine (8-OHG) is the most prevalent and of considerable importance in many human diseases (Kong and Lin, 2010; Guo et al., 2020; Li et al., 2020) due to its high affinity to pair with all bases (Li et al., 2006).

These chemically modified and oxidized adducts in mRNA lead to the generation of short polypeptides due to premature translation termination (Tanaka et al., 2007) and stalling of ribosomes (Shan et al., 2007). Structural RNAs such as tRNA also undergo cleavage and promote cell death during OS (Thompson et al., 2008; Thompson and Parker, 2009). Most importantly, angiogenin, a protein involved in blood vessel formation, directly cleaves tRNAs under stress into 5′ and 3′ halves called tiRNAs (tRNA-derived stress-induced RNAs), of which 5’ tiRNAs cause translational repression (Yamasaki et al., 2009; Ivanov et al., 2011). Moreover, it has been shown that oxidation of rRNA causes ribosome inactivation during protein synthesis (Ding et al., 2005; Honda et al., 2005). These findings suggest a link between RNA damage, dysfunction, and cell death, which may eventually lead to various diseases.

Cells may compensate for such insults by eliminating or repairing oxidatively damaged RNAs. There may be different mechanisms for eliminating different types of damages or for different RNA species. For example, human polynucleotide phosphorylase (hPNPase) preferentially binds to oxidized RNA and reduces the levels of 8-oxo-G containing RNA, thereby increasing the viability of HeLa cells under OS (Wu and Li, 2008). TruD (tRNA pseudouridine synthase) has also been shown to have high-affinity and preferential binding specificity to oxidized RNA and to protect *E. coli* cells under OS (Alluri, 2013). This suggests that proteins having preferential and high-affinity binding toward oxidized RNA may play important roles in recognizing and sequestering oxidized RNA for effective elimination. Other mechanisms of RNA quality control under OS must also exist in cells to maintain lower RNA oxidation levels. Such mechanisms may have essential functions in maintaining RNA quality and in preventing diseases related to RNA damage.

Several types of RNA granules have been observed in mammalian cells. RNA granules are spherical and nonmembranous subcellular compartments predominantly composed of RNA, RNA-binding proteins, exonucleases, helicases, ribosomal subunits, and translation factors (Moujaber and Stochaj, 2017; Anderson and Kedersha, 2006). They play an important role in the regulation of RNA localization, stability, decay, and translation. RNA granules are classified into various types based on their subcellular localization, composition, cell origin, and function, such as germinal granules (germ cells); stress granules and processing bodies (cytosol of somatic cells), nuclear paraspeckles (nucleus), and neuronal granules (neurons) (Anderson and Kedersha, 2009; Buchan, 2014).

Stress granules contain primarily untranslating mRNPs derived from mRNAs stalled in translation initiation. SGs are induced upon stress, including oxidative stress, in somatic cytosol. The formation of these SG is a survival mechanism to protect cells from stress (Moujaber and Stochaj, 2017). Besides mRNPs, numerous proteins were found in SGs, suggesting a role for SG in the induction of the innate immune response or modulation of signaling pathways (Protter and Parker, 2016). However, it has been argued that it is unlikely that the RNA components are pulled into SGs passively by the RNA-binding proteins and SGs must play active roles in RNA metabolism (Anderson and Kedersha, 2008; Wolozin and Ivanov, 2019; Advani and Ivanov, 2020). SGs were initially thought to provide protection of recruited RNAs from being damaged under stress conditions (Nover et al., 1989; Anderson and Kedersha, 2009; Lou et al., 2019; Hwang et al., 2019). More recently, it has been proposed that SGs are transition sites for inactivated mRNAs under stress, where the RNAs can be stored and be sorted for degradation or for translation reinitiation (Kedersha and Anderson, 2002; Kedersha et al., 2005; Hofmann et al., 2021). SG transcriptome studies suggest that only a subset of mRNAs are recruited into the granules without sequence preference, but with enriched longer and less actively translated mRNAs (Kedersha et al., 1999; Kedersha and Anderson, 2002; Khong et al., 2017; Khong and Parker, 2018; Protter and Parker, 2016).These findings indicate that SGs are not sites for general RNA processing but for purposes involving selected RNA molecules. It has been further suggested that RNA plays an important role in the formation and function of SGs during development and disease progression (Van Treeck and Parker, 2019; Roden and Gladfelter, 2021). However, the precise biochemical roles of SGs in RNA metabolism and function are still elusive. In this article, we critically review relevant literature and propose a hypothetical role of SG-associated proteins ADAR1, Tudor-SN, and STAU1, in control of oxidized RNA species that are potentially recruited into SGs.

### Stress Granules and Their Role in Cellular Functions and Human Diseases

SGs are membrane-less transient cytoplasmic bodies induced by various cellular stresses such as hypoxia, arsenite treatment, heat shock, oxidative stress, endoplasmic reticulum (ER)-mediated stress, and viral infections (Kedersha et al., 2000; Arimoto et al., 2008; White and Lloyd, 2012; Takahashi et al., 2013; Khong et al., 2017; Mahboubi and Stochaj, 2017; Adivarahan et al., 2018; Si et al., 2019; Tian et al., 2020). They recruit poly(A)^+^ mRNAs released from polysomes (Kedersha et al., 1999; Ivanov et al., 2019) and largely (∼50%) composed of RNA-binding proteins (Jain et al., 2016). Several important proteins/enzymes involved in RNA metabolism and translation are found in SGs, including poly(A)-binding protein (PABP) and cytotoxic granule–associated RNA-binding protein (TIA1); TIA-1-related protein (TIAR); and G3BP stress granule assembly factor 1 (G3BP1); Ago2; tristetraprolin (TTP) and HuR (Kedersha et al., 1999; Kedersha et al., 2002; Tourriere et al., 2003; Van Treeck and Parker, 2019; Tian et al., 2020; Hofmann et al., 2021). However, little is known whether and how any of these proteins or enzymes act on RNA within SGs. SGs also contain stalled preinitiation complexes, 40S ribosomal subunits, and eukaryotic initiation factors (eIF2, eIF3, eIF4A, and eIF4G) (Kimball et al., 2003; Kedersha and Anderson, 2007) along with enzymes responsible for RNA processing and decay such as exoribonuclease 1, RNA helicases, tRNA/protein ligases, tRNA/protein-methyltransferases, RNA-specific adenosine deaminases, phosphatases, and kinases (Jain et al., 2016; Van Treeck and Parker, 2019). Cell signaling factors such as mTORC1 are found in SGs induced by oxidative stress, suggesting a role for these SGs in modulating signal pathways (Wippich et al., 2013). SGs induced by virus infection recruit and activate many antiviral proteins, such as RIG-1, PKR, OAS, and RNase L, enhancing innate immune response and viral resistance (Onomoto et al., 2014; Protter and Parker, 2016; Yoneyama et al., 2016; Gao et al., 2021). SGs are also induced by inosine-modified RNA, overexpression of translational repressors, and angiogenin-induced tiRNAs (tRNA-derived stress-induced RNA fragments) (Scadden, 2007; Mollet et al., 2008; Emara et al., 2010), etc. These SGs transiently repress translation by sequestration of mRNAs (Mollet et al., 2008; Souquere et al., 2009).

Recent studies demonstrated that membrane-less organelles may arise through a process called liquid–liquid phase separation (LLPS). It is a reversible process of a homogeneous mixture into a dilute and condensed phase (Dolgin, 2018; Li et al., 2020). There is an increasing evidence, demonstrating that SGs are generated from LLPS process (Brangwynne et al., 2009; Li et al., 2012; Wippich et al., 2013; Hyman et al., 2014; Molliex et al., 2015; Patel et al., 2015; Alberti et al., 2019; Hofmann et al., 2021). RNA and RNA-binding proteins are essential for LLPS and condense into liquid droplets during phase separation (Alberti et al. (2019)) and these components become concentrated into discrete loci (Hyman et al., 2014). RNA–RNA interactions also cause phase separation *in vitro* and possibly contribute to SG formation (Van Treeck and Parker, 2019). RNA not only actively contributes to the formation of molecular condensates in LLPS, but may play important roles in modulating the function of the condensates in cells’ physiological and pathological processes (Roden and Gladfelter, 2021).

It is well known that acute and destructive oxidative stress can lead to cell death/apoptosis. Arsenite treatment, heat shock, and hypoxia-induced OS and SGs formed under these conditions were shown to inhibit apoptosis (Arimoto et al., 2008; Takahashi et al., 2013; Si et al., 2019). Moreover, mutations in TDP-43 led to a significant reduction in SG formation and increased apoptosis in human fibroblasts (Orrù et al., 2016). SGs suppress ROS generation (Takahashi et al., 2013) and attenuate RACK1 (Park et al., 2020), which may be part of the mechanism for apoptosis inhibition. Another possible role of SGs under OS may involve the elimination of damaged cellular macromolecules including oxidized RNA, which will be discussed below.

Recent evidence suggests that SG-enriched ATF4 mRNA translates efficiently within SGs (Mateju et al., 2020) and U2OS cells deficient in making SGs exhibited stress-induced translational repression (Kedersha et al., 2016), suggesting that SGs are not only required for translational arrest but also for many cellular functions yet unknown. These studies demonstrate that SGs are generated as a defense mechanism to protect cells against adverse effects of various stresses.

Abnormality and deficiency of SGs are implicated in various human diseases such as promoting cancer cell survival and tumor progression (Anderson et al., 2015; Somasekharan et al., 2015; Grabocka and Bar-Sagi, 2016; Van Treeck and Parker, 2019) and in the pathogenesis of degenerative diseases such as amyotrophic lateral sclerosis (ALS), Alzheimer’s disease (AD), and frontotemporal dementia (FTD) (Li et al., 2013; Ramaswami et al., 2013; Molliex et al., 2015). Recent studies also showed that SGs are involved in vascular injury and atherosclerosis (Herman et al., 2019). Additionally, SGs create an antiviral state by regulating viral replication and immune response (Valiente-Echeverria et al., 2012; Poblete-Durán et al., 2016; McCormick and Khaperskyy, 2017; Van Treeck and Parker, 2019; Tian et al., 2020). Recent evidence suggested that endoribonuclease nsp15 from SARS-CoV-2 interfered with SG formation and evaded sequestration of viral components in SGs (Gao et al., 2021).

### Stress Granules Are Enriched with mRNAs Containing Long 3′ UTRs with an Inverted Repeat *Alus*

SGs and RNA granules induced by OS and other stresses are highly and specifically enriched with mRNA transcripts containing long 3′ UTRs and poly(A) tails from human cells (Kato et al., 2012; Weissbach and Scadden, 2012). The average length of 3′ UTR in mRNA found in SGs is 2.18 ± 0.81 Kb (Han et al., 2012). Poly(A)-binding protein 1 (PABP1) binds to poly(A) tails in mRNA and brings other RNA-binding proteins into SGs. Indeed, it has been reported that reporter mRNAs with long 3′ UTRs harboring IR Alus accumulate in SGs, whereas mRNAs with either a single sense *Alu*, antisense *Alu,* or without any *Alu* in their 3′ UTR diffuse throughout the cytoplasm (Fitzpatrick and Huang, 2012; Weissbach and Scadden, 2012). Other studies also demonstrated RNA granules are enriched with RNA with long 3′ UTRs (Han et al., 2012; Kato et al., 2012). Analysis of length and translatability features of RNA species revealed that longer mRNA and ncRNA transcripts (an average length of 7.1 and 1.9 kb, respectively) and mRNAs with poor translatability are enriched in SGs generated by arsenite-induced oxidative stress in human cells (Khong et al., 2017). Results of deep sequencing studies in human cells and mouse brain tissue demonstrated that RNA species containing long 3′UTRs are highly enriched in RNA granule fractions compared to granule-depleted cytoplasmic fractions (Han et al., 2012). Additionally, recent studies revealed that endoplasmic reticulum stress-induced RNA granules are enriched with a subset of translationally suppressed mRNAs characterized by extended transcript length and AU-rich motifs (Namkoong et al., 2018). However, the precise reason for specific recruitment and accumulation of long 3′ UTR-containing mRNAs into SGs is still unknown.

Interestingly, the heat-shock proteins Hsp70 and Hsp90 and their respective mRNAs that are preferentially expressed under cellular stress are excluded from SGs (Stohr et al., 2006; Anderson and Kedersha, 2009). The mechanism by which these molecules are excluded from SGs is still elusive (Anderson and Kedersha, 2009). It is striking to note that heat shock-induced alternative polyadenylation in hsp70 transcripts leads to a shortened 3′ UTR and loss of the miR-378* binding site (Tranter et al., 2011). It appears that in order for the hsp70 transcript to be translated and available during stress conditions, it should neither be recruited to SGs, nor be a target for miRNA. For this very reason, the hsp70 transcript undergoes alternative polyadenylation leading to shortening of the 3′UTR and loss of the miR-378* binding site. This is an example of controlled SG recruitment by regulating the length of 3′ UTR. Interestingly, ischemia, a stressful condition in the murine heart, is accompanied by decreased levels of miR-378* (Tranter et al., 2011; Knezevic et al., 2012).

In addition to the RNA species described above, SGs are also enriched in poly(A)^+^ mRNA (Kedersha et al., 1999; Kedersha et al., 2002; Souquere et al., 2009). However, only 50% of the cytoplasmic poly(A)-containing transcripts are recruited to SGs in mammalian cells, indicating selective mRNA recruitment (Kedersha et al., 1999; Kedersha and Anderson, 2002).

Mounting evidence suggests that long UTRs in mRNAs are indispensable for the mRNAs to be recruited into SGs via LLPS. Recent studies demonstrated that tandem binding of 3′ UTR of p53 mRNA by TIA-1 (an abundant SG protein) efficiently enhanced phase separation and formed a potential site for SG assembly (Loughlin et al., 2021). Deb1p is an RNA helicase that promotes translation of long mRNAs with highly structured 5′ UTRs (Sen et al. (2015); Guenther et al., 2018); Iserman et al. (2020) reported that the budding yeast Deb1p was condensed into SGs upon heat shock *via* the LLPS process and the protein’s activity was inhibited. Deb1p condensation led to translational repression of mRNAs with structurally complex 5′ UTRs (Iserman et al., 2020). Interestingly, the mRNAs encoding heat-shock proteins (hsps) have short and unstructured 5′ UTRs and evade translational repression by Deb1p condensation (Iserman et al., 2020). These studies demonstrate that SGs are enriched with long mRNAs having complex structured 5′ and 3′ UTRs and are mostly devoid of RNA species with short UTRs.

### Interaction of Stress Granule and Processing Body

Processing bodies are cytoplasmic granules composed of mRNA binding decapping enzymes along with exonucleases and are enriched with mRNA species similar to SGs, but without poly(A) tails (Zheng et al., 2008; Aizer et al., 2014; Ivanov et al., 2019). P-bodies play an important role in the regulation of RNA translation, storage, and degradation (Sheth and Parker, 2003; Sheth and Parker, 2006; Buchan et al., 2008; Hubstenberger et al., 2017; Matheny et al., 2019). Findings from several laboratories have revealed a physical interaction between SGs and PBs upon stress induction, a phenomenon called docking. Interestingly, SGs and PBs are formed simultaneously in response to oxidative stress, and nearly all PBs were found together and in physical association with SGs *in vivo* (Kedersha et al., 2005). Importantly, both SGs and PBs were found to contain similar mRNA species (Kedersha et al., 2005), suggesting the exchange of mRNA between these organelles. It is intriguing that most of the mRNAs in SGs are poly(A) enriched (Kedersha and Anderson, 2002; Souquere et al., 2009), but the majority of mRNAs present in PBs are either devoid of or have only a short oligo(A) tail (Zheng et al., 2008; Aizer et al., 2014; Ivanov et al., 2019), indicating that poly(A)+ mRNAs in SGs were processed before transferring to PBs. This finding supports the hypothesis of Kedersha et al. that “mRNAs destined for decay are sorted in stress granules and subsequently transported into processing (P) bodies.”

### Role of RNA Editing Enzymes in Stress Granules

Recent findings demonstrate that the p150 isoform of adenosine deaminase acting on RNA (ADAR1) and the dsRNA-binding protein Tudor staphylococcal nuclease (Tudor-SN) are specifically recruited and colocalized in SGs upon OS induction (Weissbach and Scadden, 2012). A specific role of Tudor-SN is discussed in the section below. Under normal physiological conditions, ADAR1 deaminates and converts adenosine to inosine (A– I) in dsRNA regions of 3′ UTRs. In addition to its presence in OS-induced SGs, ADAR1 is also localized in measles virus-induced SGs (Okonski and Samuel, 2013). IR *Alus* accumulated in SGs form double-stranded structures in the mRNA 3’ UTR regions which may provide higher stability and become targets for ADARs (Kim et al., 2004). Weissbach and Scadden (2012) speculated that ADAR1-p150 may edit a subset of mRNAs within SGs, resulting in the generation of specific I-U-dsRNA. In support of this assumption, previous studies have shown that inosine-containing dsRNAs are specifically bound to SG-like complexes (Scadden, 2007), suggesting a role for ADAR1-dependent editing in SGs.

Additional evidence supports the recruitment of ADAR1-p150 specifically in SGs under hypoxia- or arsenite-induced OS. Upon introduction of hypoxia, ADAR1-p150 isoform levels were elevated 3.4-fold (Nevo-Caspi et al., 2011). In contrast, the level of ADAR1-p110 isoform was unaffected. Interestingly, when cells were treated with arsenite to induce OS, only the ADAR1-p150 isoform, but not ADAR1-p110, was recruited to SGs (Weissbach and Scadden, 2012). The p110 isoform is a truncated form of p150 lacking a Z-DNA/RNA binding domain (ZαADAR1) at the N-terminus. The p110 isoform contains a nuclear export signal (NES) and is exclusively found in the nucleus, whereas p150 is present in the cytoplasm where it edits double-stranded regions of 3′ UTRs (George and Samuel, 1999; Poulsen et al., 2001; Christofi and Zaravinos 2019; Lamers et al., 2019). Subsequent studies demonstrated that ZαADAR1 is essential and is the sole determinant for p150’s localization to SGs (Ng et al., 2013; Chiang et al., 2021). Consistent with elevated ADAR1-p150 under hypoxia, *MED13*, *STAT3*, and *F11R* transcripts which contain IR *Alus* were found to have mostly elevated A–I editing levels (Nevo-Caspi et al., 2011), although this study did not report the localization of these transcripts in hypoxia-induced SGs. More recent transcriptome studies of arsenite-induced SGs revealed that *MED13*, *STAT3*, and *F11R* transcripts are indeed localized in SGs, supporting the concept that IR *Alu*-containing transcripts are recruited to SGs (Khong et al., 2017). These studies suggest that ADAR1-p150 is induced upon stress followed by specific recruitment to SGs and there it may possibly alter A–I editing of mRNA transcripts having IR *Alus* (Weissbach and Scadden, 2012). It is worth noting that recently rapid progress in SG studies has greatly expanded our knowledge about the various aspects of SGs. However, little attention was paid to the role of ADAR1-p150 in SGs since it was last reported almost 10 years ago. The ability of ADAR1-p150 to edit mRNA transcripts in SGs has not yet been demonstrated experimentally, and the downstream effects of potential editing in these transcripts in SGs remain unknown. It is important to explore the role of ADAR1-p150 in SGs from new angles.

Although the role of ADAR1-edited dsRNAs has been implicated in various cellular functions, their role in SGs is still unknown. Several studies have demonstrated the relationship between RNA editing and interferon stimulation. Interferons (INFs) induce ADAR1 (Strehblow et al., 2002; Herbert 2019). On the other hand, ADAR1-edited IU-dsRNAs were found to inhibit poly(IC)-induced apoptosis, viral RNA stimulation, and INF production (Vitali and Scadden, 2010; Yang et al., 2014; Liddicoat et al., 2015; Yu et al., 2015; Wang et al., 2017). These studies demonstrated that IU-dsRNAs are anti-inflammatory and inhibit interferon responses to dsRNA (Mannion et al., 2014). Interestingly, previous studies have shown that inosine-containing dsRNAs (I-dsRNA) are specifically bound to SG-like complexes (Scadden, 2007), suggesting a role for ADAR1-dependent editing in SGs. It remains to be determined whether SG-mediated RNA editing, if it happens, plays a role in interferon response, or in some other processes.

### Role of Tudor-Staphylococcal Nuclease in Stress Granules

The Tudor-staphylococcal nuclease (Tudor-SN) is a multifunctional protein with major implications in SG assembly, gene regulation, and pre-mRNA splicing (Yang et al., 2007; Gao et al., 2010; Gao et al., 2012). Additionally, Tudor-SN also emerged as a novel poly(A) mRNA–binding protein which colocalizes with PABP1, a marker of stress granule (Gao et al., 2015), and modulates the kinetics of angiotensin II receptor, type 1 mRNA-3′UTR aggregation in SGs (Gao et al., 2014). *In vitro* studies revealed that Tudor-SN specifically binds to and cleaves runs of I–U and U–I rich regions in dsRNA (Scadden, 2005), demonstrating that ADAR1-edited dsRNAs are ideal for Tudor-SN cleavage (Hundley and Bass, 2010). As discussed above, ADAR1-edited dsRNAs may be enriched in 3′UTRs of mRNAs in SGs. Tudor-SN cleavages may result in removal of the poly(A) tail and/or shortening of 3′ UTRs, initiating degradation of these mRNAs. In contrast, Tudor-SN cleavage of endogenous transcripts under normal physiological conditions has not been detected; however, the nuclease can cleave inosine-containing dsRNA in response to certain environmental stimuli (Hundley and Bass, 2010). While an endogenous inosine-containing mRNA, CTN–RNA was cleaved at its 3′ UTR in response to stress (Prasanth et al., 2005), the involvement of Tudor-SN was not demonstrated. Recent studies have shown that Tudor-SN functions as a nucleocytoplasmic shuttling protein associated with poly(A)-containing mRNAs that is involved in their trafficking in and out of SGs and the nucleus (Gao et al., 2015). This suggests that both ADAR1-p150 and Tudor-SN coordinate in SGs to process a specific set of mRNAs. However, delineating the precise biochemical functions and consequences of ADAR1-p150 and Tudor-SN actions in SGs will require further study.

### Role of Staufen 1 in Stress Granule

Staufen 1 (STAU1) is a double-stranded RNA-binding protein associated with polysomes and recruited to SGs upon OS. It is always present in SGs during their assembly and dissolution; however, it is not required for SG formation (Thomas et al., 2005; Thomas et al., 2009). In addition, STAU1 is involved in mRNA transport in both somatic cells and oocytes of vertebrates and invertebrates (Ferrandon et al., 1994; Broadus et al., 1998; Kiebler et al., 1999; Micklem et al., 2000; Tang et al., 2001). STAU1 binds and coprecipitates with mRNAs having inverted *Alu* repeats in their 3′ UTRs and prevents nuclear retention and promotes export of the mRNAs to the cytoplasm (Elbarbary et al., 2013). Further studies found that STAU1-bound transcripts have 3–4-fold longer 3′ UTRs compared to unbound transcripts (Laver et al., 2013). Elbarbary et al. have demonstrated that underedited reporter mRNAs with IR *Alus* inhibit STAU1-mediated export, suggesting the requirement for A–I hyperediting for STAU1-mediated export of such mRNAs. It has been shown that the mammalian STAU1, when bound to 3′UTR, triggers mRNA decay mediated by UPF1, an important factor involved in the degradation of nonsense mRNA (Kim et al., 2005). Recent studies have also demonstrated that STAU1-mediated mRNA decay (SMD) targets RNA–RNA duplexes formed between the *Alu* repeat in the 3′-UTR of one mRNA and another *Alu* repeat in long noncoding RNA (lncRNA) (Gong and Maquat, 2011; Park and Maquat, 2013). These studies suggest that STAU1 preferably binds to long 3′ UTRs having IR *Alus* and selectively exports hyperedited transcripts.

A strong relationship has been established between SGs and STAU1. Stabilization of polysomes by cycloheximide prevented the formation of SGs (Thomas et al., 2009) due to retention of RNA in polysomes, suggesting a requirement for polysome-free mRNAs for SG assembly. Findings from the same laboratory also revealed that knockdown of STAU1-enhanced SG formation and accumulation (Thomas et al., 2009), while the stability of SGs mostly depends upon the amount of accumulated mRNP complex. Hypothetically, this could be due to the fact that STAU1 being a natural exporter of RNA molecules with a specific affinity toward a subset of mRNAs with long 3’ UTRs, and IR *Alus* and STAU1 depletion prevents export of these RNAs to PBs, resulting in stabilization and accumulation of SGs. In contrast, overexpression of STAU1 resulted in inhibition of SG accumulation perhaps due to STAU1-mediated rapid export of mRNA from SGs to PBs. Endogenous STAU1 was barely detected in PBs under resting conditions, though upon stress induction it was recruited to PBs (Thomas et al., 2009). When overexpressed, STAU1 is sporadically detected in PBs under resting conditions, but upon induction of OS, the proportion of STAU1-containing PBs increases (Thomas et al., 2009), suggesting that a portion of STAU1 is specifically recruited to PBs under OS. These studies suggest that STAU1 facilitates the export of mRNAs from SGs to PBs, thereby destabilizing SGs. However, detailed studies must be performed in order to prove this concept.

### Oxidized RNAs Accumulate in Stress Granules

Several pieces of evidence support a notion that oxidized RNA is recruited to SGs or similar RNA granules. First, 8-OHG-containing RNAs were observed in “oxidized RNA bodies (ORBs)” within living HeLa cells by staining with 8-OHG-specific antibody (Zhan et al., 2015). It is likely that these ORBs are related to or can interact with SGs. Recent evidence further demonstrates that several mitochondrial tRNAs and 5S rRNA, which are presumably highly oxidized in this ROS-generating organelle, are localized in SGs (Khong et al., 2017). Oxidative damage to RNA can also lead to abasic sites (apurinic/apyrimidinic sites). Unpublished observations from Pourkalbassi, Lu, and Li revealed that in HeLa cells, abasic RNA accumulates in H_2_O_2_-induced SGs but not in P-bodies. Furthermore, the proteins that bind specifically to oxidized RNA such as human PNPase and PCBP1 are reported to localize in SGs (Jain et al., 2016; Ishii et al., 2018; Markmiller et al., 2018). These findings suggest that SGs or related RNA foci may recruit oxidized RNAs involving specific protein factors and protect cells from adverse effects of oxidized RNA.

Presently, little is known about the identity of abasic RNAs in H_2_O_2_-induced SGs or 8-OHG-containing RNAs in ORBs. It is likely that highly structured RNA species are preferentially oxidized. *In vivo* and *in vitro* studies have demonstrated that noncoding structural RNAs such as tRNA and rRNA molecules having complex double-stranded structures are oxidized to a greater extent in their native conformation compared to their denatured forms (Liu, 2012; Liu et al., 2012; Liu et al., 2020). Moreover, double-stranded RNA:DNA duplexes are oxidized to a greater extent than single-stranded RNA molecules (Liu et al., 2020). Whether oxidized, highly structured RNAs are recruited to SGs for elimination remains to be studied.

Consistent with the above mentioned notion that a selected set of RNAs are recruited in SGs, it was found that a subset of mRNAs is subject to high oxidative damage. Immunoprecipitation assays combined with sequence analysis revealed enrichment of 8-OHG in a subset of mRNAs in Alzheimer’s brains (Shan et al., 2003) or OS-treated yeast cultures (McKinlay et al., 2012). This could provide a possible explanation for the accumulation of mRNA molecules with long 3’ UTRs, IR *Alus,* or AREs, with complex double-stranded structures in SGs. It is likely that such mRNAs are more subject to oxidative damages, especially under OS, and are specifically enriched in SGs. From all the findings described above, it appears that RNA molecules that are oxidized and prone to oxidative damages are more likely to be recruited to SGs.

## Hypothesis

**FIGURE 1:**
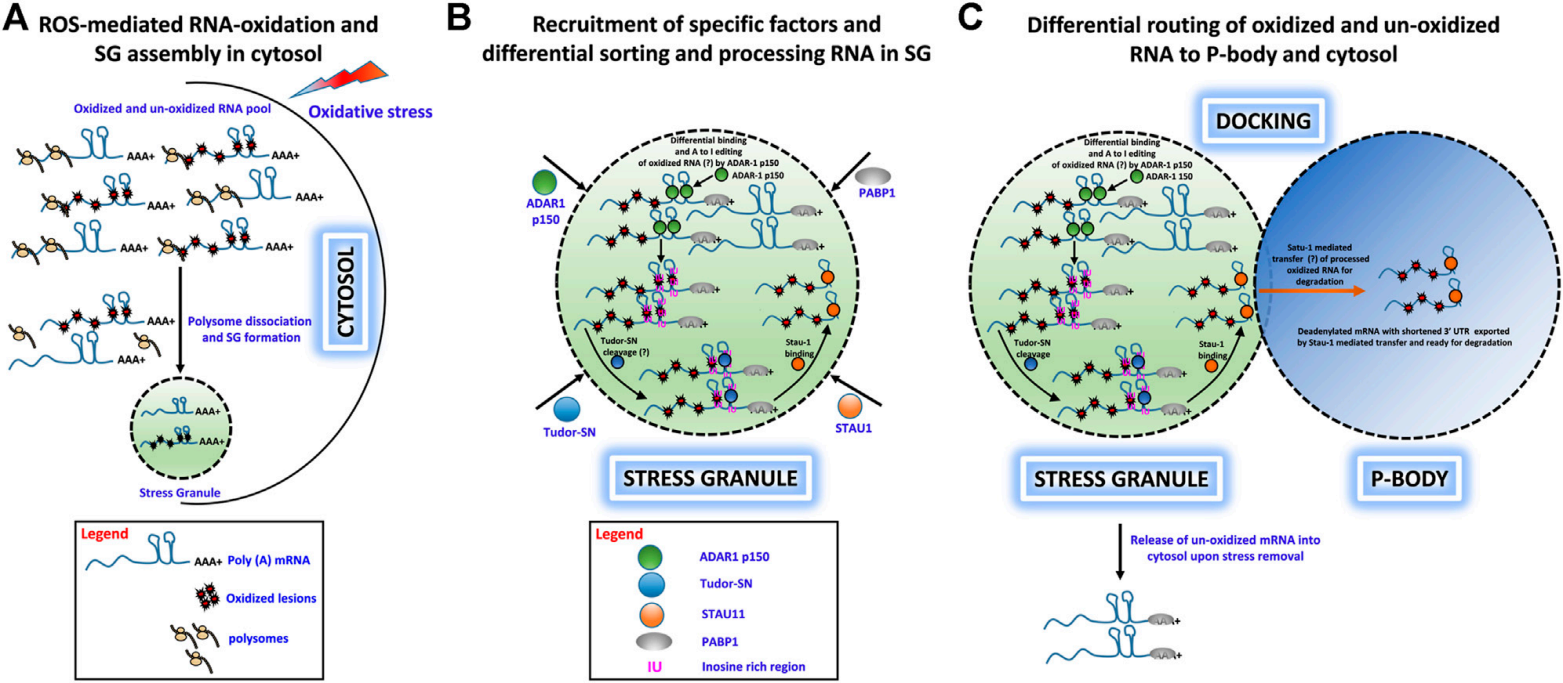
Hypothetical role of ADAR1, Tudor-SN, and STAU1 in stress granule-mediated oxidized RNA decay. **(A)** ROS-mediated oxidation of mRNA molecules and stress granule formation under oxidative stress in the cytosol. Polysome-dissociated mRNA molecules with long 3′ UTRs along with several protein factors accumulate and form membrane-free stress granules. **(B)** Specific recruitment of protein factors ADAR1, Tudor-SN, and STAU1 to stress granules. Under oxidative stress, ADAR1 and Tudor-SN translocate to stress granules specifically, along with STAU1. ADAR1 may preferentially hyperedit (A– I) in ds 3′ UTR regions, and Tudor-SN cleaves mRNA at IU/oxidative lesion-rich regions to produce mRNA with short 3′ UTRs. These shortened mRNAs devoid of a poly-A tail are bound by STAU1 for subsequent processing. **(C)** Differential routing of oxidized and unoxidized mRNA from SG. Under oxidative stress, stress granules and P-bodies physically interact. During this docking process, STAU1 in SG binds to Tudor-SN processed, oxidized mRNA and transports it to P-bodies for degradation. Additionally, upon removal of oxidative stress, unoxidized and sequestered mRNA may be released from the stress granules for translational reinitiation.

We have proposed a hypothetical model to describe how under various physiological stress conditions (e.g. oxidative stress) a specific set of RNA molecules are damaged by oxidative stress insults and traffic to SGs for sequestration. From there, a subset of these RNAs is shuttled to PBs for degradation. This process protects cells from adverse consequences of RNA oxidation and enhances cell survival. This hypothesis is captured in the following key points:

• Under oxidative stress conditions, cells generate excessive ROS, which cause extensive chemical and physical damage to RNA molecules and can lead to premature termination of translation and cytotoxicity. Cells have developed mechanisms to eliminate such damaged RNAs presumably by rapid degradation (Figure 1A)*.*
• Upon induction of OS, free mRNAs with long 3′ UTRs resulting from polysome dissociation promote SG assembly, and both oxidatively damaged and nonoxidized mRNAs are routed to SGs. ADAR1-p150 is recruited to SGs along with Tudor-SN, where ADAR1-p150 sorts oxidized and unoxidized mRNAs and preferentially binds to oxidatively damaged RNAs in their double-stranded 3′ UTR regions and converts A–I, which then forms the substrate for Tudor-SN cleavage (Figure 1B).
• Tudor-SN cleaves inosine-containing dsRNA resulting in shortening of 3′ UTR or loss of the poly(A) tail, and this forms the basis for mRNA decay in PBs. Apart from ADAR1 and Tudor-SN, Staufen1 (STAU1) is also recruited to SG upon OS induction. STAU1 is a natural transporter of mRNA molecules, and it can bind to the 3′UTR of Tudor-SN processed mRNA and transport it from SGs to PBs where it is degraded (Figures 1B,C).
• We speculate that oxidized RNAs are recognized and sequestered in the SG-mediated degradation process. First, ADAR1-p150 may preferentially recognize and edit oxidatively damaged mRNA and that these RNAs will be sorted and routed specifically to PBs for degradation, while normal mRNAs may be released from SGs for reinitiation of translation upon stress removal. Second (or alternatively), oxidized RNAs may contain oxidatively damaged nucleobases that behave like edited bases and trigger selected cleavage by Tudor-SN-like activities (Figures 1A–C).

## Data Availability

The original contributions presented in the study are included in the article/Supplementary Material; further inquiries can be directed to the corresponding author/s.

## References

[B1] Adivarahan S. Livingston N. Nicholson B. Rahman S. Wu B. Rissland O. S. (2018). Spatial Organization of Single mRNPs at Different Stages of the Gene Expression Pathway. Mol. Cel 72, 727–738. 10.1016/j.molcel.2018.10.010 PMC6592633 30415950

[B2] Advani V. M. Ivanov P. (2020). Stress Granule Subtypes: an Emerging Link to Neurodegeneration. Cell. Mol. Life Sci. 77 (23), 4827–4845. 10.1007/s00018-020-03565-0 32500266 PMC7668291

[B3] Aizer A. Kalo A. Kafri P. Shraga A. Ben-Yishay R. Jacob A. (2014). Quantifying mRNA Targeting to P-Bodies in Living Human Cells Reveals Their Dual Role in mRNA Decay and Storage. J. Cel Sci 127 (20), 4443–4456. 10.1242/jcs.152975 25128566

[B4] Alberti S. Gladfelter A. Mittag T. (2019). Considerations and Challenges in Studying Liquid-Liquid Phase Separation and Biomolecular Condensates. Cell 176 (3), 419–434. 10.1016/j.cell.2018.12.035 30682370 PMC6445271

[B5] Alluri R. K. (2013). tRNA Processing and Quality Control in Bacteria. [Ph.D dissertation]. Boca Raton, FL: Florida Atlantic University. Publication # 3571440.

[B6] Anderson P. Kedersha N. Ivanov P. (2015). Stress Granules, P-Bodies and Cancer. Biochim. Biophys. Acta (Bba) - Gene Regul. Mech. 1849, 861–870. 10.1016/j.bbagrm.2014.11.009 PMC4457708 25482014

[B7] Anderson P. Kedersha N. (2006). RNA Granules. J. Cell Biol. 172, 803–808. 10.1083/jcb.200512082 16520386 PMC2063724

[B8] Anderson P. Kedersha N. (2009). RNA Granules: post-transcriptional and Epigenetic Modulators of Gene Expression. Nat. Rev. Mol. Cell. Biol. 10, 430–436. 10.1038/nrm2694 19461665

[B9] Anderson P. Kedersha N. (2008). Stress Granules: the Tao of RNA Triage. Trends Biochem. Sci. 33 (3), 141–150. 10.1016/j.tibs.2007.12.003 18291657

[B10] Arimoto K. Fukuda H. Imajoh-Ohmi S. Saito H. Takekawa M. (2008). Formation of Stress Granules Inhibits Apoptosis by Suppressing Stress-Responsive MAPK Pathways. Nat. Cell Biol. 10, 1324–1332. 10.1038/ncb1791 18836437

[B11] Bae Y. S. Oh H. Rhee S. G. Yoo Y. D. (2011). Regulation of Reactive Oxygen Species Generation in Cell Signaling. Mol. Cell 32 (6), 491–509. 10.1007/s10059-011-0276-3 PMC3887685 22207195

[B12] Barciszewski J. Barciszewska M. Z. Siboska G. Rattan S. I. S. Clark B. F. C. (1999). Some Unusual Nucleic Acid Bases Are Products of Hydroxyl Radical Oxidation of DNA and RNA. Mol. Biol. Rep. 26, 231–238. 10.1023/a:1007058602594 10634505

[B13] Brand M. D. (2010). The Sites and Topology of Mitochondrial Superoxide Production. Exp. Gerontol. 45, 466–472. 10.1016/j.exger.2010.01.003 20064600 PMC2879443

[B14] Brangwynne C. P. Eckmann C. R. Courson D. S. Rybarska A. Hoege C. Gharakhani J. (2009). Germline P Granules Are Liquid Droplets that Localize by Controlled Dissolution/condensation. Science 324, 1729–1732. 10.1126/science.1172046 19460965

[B15] Broadus J. Fuerstenberg S. Doe C. Q. (1998). Staufen-dependent Localization of Prospero mRNA Contributes to Neuroblast Daughter-Cell Fate. Nature 391, 792–795. 10.1038/35861 9486649

[B16] Buchan J. R. (2014). mRNP Granules. RNA Biol. 11, 1019–1030. 10.4161/15476286.2014.972208 25531407 PMC4615263

[B17] Buchan J. R. Muhlrad D. Parker R. (2008). P Bodies Promote Stress Granule Assembly in *Saccharomyces cerevisiae* . J. Cell Biol 183, 441–455. 10.1083/jcb.200807043 18981231 PMC2575786

[B18] Chang Y. Kong Q. Shan X. Tian G. Ilieva H. Cleveland D. W. (2008). Messenger RNA Oxidation Occurs Early in Disease Pathogenesis and Promotes Motor Neuron Degeneration in ALS. PLoS ONE 3 (8), e2849. 10.1371/journal.pone.0002849 18682740 PMC2481395

[B19] Chiang C. Li Y. Ng S. K. (2021). The Role of the Z-DNA Binding Domain in Innate Immunity and Stress Granules. Front. Immunol. 11, 625504. 10.3389/fimmu.2020.625504 33613567 PMC7886975

[B20] Christofi T. Zaravinos A. (2019). RNA Editing in the Forefront of Epitranscriptomics and Human Health. J. Transl Med. 17 (1), 319. 10.1186/s12967-019-2071-4 31547885 PMC6757416

[B21] Ding Q. Markesbery W. R. Chen Q. Li F. Keller J. N. (2005). Ribosome Dysfunction Is an Early Event in Alzheimer's Disease. J. Neurosci. 25, 9171–9175. 10.1523/jneurosci.3040-05.2005 16207876 PMC6725754

[B22] Dolgin E. (2018). What Lava Lamps and Vinaigrette Can Teach Us about Cell Biology. Nature 555 (7696), 300–302. 10.1038/d41586-018-03070-2 29542707

[B23] Dröge W. (2002). Free Radicals in the Physiological Control of Cell Function. Physiol. Rev. 82, 47–95. 10.1152/physrev.00018.2001 11773609

[B24] Elbarbary R. A. Li W. Tian B. Maquat L. E. (2013). STAU1 Binding 3' UTR IRAlus Complements Nuclear Retention to Protect Cells from PKR-Mediated Translational Shutdown. Genes Dev. 27, 1495–1510. 10.1101/gad.220962.113 23824540 PMC3713430

[B25] Emara M. M. Ivanov P. Hickman T. Dawra N. Tisdale S. Kedersha N. (2010). Angiogenin-induced tRNA-Derived Stress-Induced RNAs Promote Stress-Induced Stress Granule Assembly*. J. Biol. Chem. 285, 10959–10968. 10.1074/jbc.m109.077560 20129916 PMC2856301

[B26] Ferrandon D. Elphick L. Nüsslein-Volhard C. St Johnston D. (1994). Staufen Protein Associates with the 3′UTR of Bicoid mRNA to Form Particles that Move in a Microtubule-dependent Manner. Cell 79, 1221–1232. 10.1016/0092-8674(94)90013-2 8001156

[B27] Fitzpatrick T. Huang S. (2012). 3'-UTR-located Inverted Alu Repeats Facilitate mRNA Translational Repression and Stress Granule Accumulation. Nucleus 3, 359–369. 10.4161/nucl.20827 22688648 PMC3679280

[B28] Gao B. Gong X. Fang S. Weng W. Wang H. Chu H. (2021). Inhibition of Anti-viral Stress Granule Formation by Coronavirus Endoribonuclease Nsp15 Ensures Efficient Virus Replication. Plos Pathog. 17 (2), e1008690. 10.1371/journal.ppat.1008690 33635931 PMC7946191

[B29] Gao X. Fu X. Song J. Zhang Y. Cui X. Su C. (2015). Poly(A)+mRNA-binding Protein Tudor-SN Regulates Stress Granules Aggregation Dynamics. FEBS J. 282 (5), 874–890. 10.1111/febs.13186 25559396

[B30] Gao X. Ge L. Shao J. Su C. Zhao H. Saarikettu J. (2010). Tudor-SN Interacts with and Co-localizes with G3BP in Stress Granules under Stress Conditions. FEBS Lett. 584, 3525–3532. 10.1016/j.febslet.2010.07.022 20643132 PMC7127458

[B31] Gao X. Shi X. Fu X. Ge L. Zhang Y. Su C. (2014). Human Tudor Staphylococcal Nuclease (Tudor-SN) Protein Modulates the Kinetics of AGTR1-3′UTR Granule Formation. FEBS Lett. 588, 2154–2161. 10.1016/j.febslet.2014.04.045 24815690

[B32] Gao X. Zhao X. Zhu Y. He J. Shao J. Su C. (2012). Tudor Staphylococcal Nuclease (Tudor-SN) Participates in Small Ribonucleoprotein (snRNP) Assembly via Interacting with Symmetrically Dimethylated Sm Proteins. J. Biol. Chem. 287, 18130–18141. 10.1074/jbc.m111.311852 22493508 PMC3365748

[B33] George C. X. Samuel C. E. (1999). Human RNA-specific Adenosine Deaminase ADAR1 Transcripts Possess Alternative Exon 1 Structures that Initiate from Different Promoters, One Constitutively Active and the Other Interferon Inducible. Proc. Natl. Acad. Sci. 96, 4621–4626. 10.1073/pnas.96.8.4621 10200312 PMC16382

[B34] Gong C. Maquat L. E. (2011). lncRNAs Transactivate STAU1-Mediated mRNA Decay by Duplexing with 3′ UTRs via Alu Elements. Nature 470, 284–288. 10.1038/nature09701 21307942 PMC3073508

[B35] Grabocka E. Bar-Sagi D. (2016). Mutant KRAS Enhances Tumor Cell Fitness by Upregulating Stress Granules. Cell 167, 1803–1813. 10.1016/j.cell.2016.11.035 27984728 PMC5441683

[B36] Guenther U.-P. Weinberg D. E. Zubradt M. M. Tedeschi F. A. Stawicki B. N. Zagore L. L. (2018). The Helicase Ded1p Controls Use of Near-Cognate Translation Initiation Codons in 5′ UTRs. Nature 559, 130–134. 10.1038/s41586-018-0258-0 29950728 PMC6226265

[B37] Guo C. Chen Q. Chen J. Yu J. Hu Y. Zhang S. (2020). 8-Hydroxyguanosine as a Possible RNA Oxidative Modification Marker in Urine from Colorectal Cancer Patients: Evaluation by Ultra Performance Liquid Chromatography-Tandem Mass Spectrometry. J. Chromatogr. B 1136, 121931. 10.1016/j.jchromb.2019.121931 31855840

[B38] Han T. W. Kato M. Xie S. Wu L. C. Mirzaei H. Pei J. (2012). Cell-free Formation of RNA Granules: Bound RNAs Identify Features and Components of Cellular Assemblies. Cell 149, 768–779. 10.1016/j.cell.2012.04.016 22579282

[B39] Herbert A. (2019). Z-DNA and Z-RNA in Human Disease. Commun. Biol. 2 (1), 7. 10.1038/s42003-018-0237-x 30729177 PMC6323056

[B40] Herman A. B. Silva Afonso M. Kelemen S. E. Ray M. Vrakas C. N. Burke A. C. (2019). Regulation of Stress Granule Formation by Inflammation, Vascular Injury, and Atherosclerosis. Atvb 39 (10), 2014–2027. 10.1161/atvbaha.119.313034 PMC6761003 31462091

[B41] Hofer T. Badouard C. Bajak E. Ravanat J. L. Mattsson A. Cotgreave I. A. (2005). Hydrogen Peroxide Causes Greater Oxidation in Cellular RNA Than in DNA. Biol. Chem. 386, 333–337. 10.1515/bc.2005.040 15899695

[B42] Hofmann S. Kedersha N. Anderson P. Ivanov P. (2021). Molecular Mechanisms of Stress Granule Assembly and Disassembly. Biochim. Biophys. Acta (Bba) - Mol. Cell Res. 1868 (1), 118876. 10.1016/j.bbamcr.2020.118876 PMC7769147 33007331

[B43] Honda K. Smith M. A. Zhu X. Baus D. Merrick W. C. Tartakoff A. M. (2005). Ribosomal RNA in Alzheimer Disease Is Oxidized by Bound Redox-Active Iron*. J. Biol. Chem. 280, 20978–20986. 10.1074/jbc.m500526200 15767256

[B44] Hubstenberger A. Courel M. Bénard M. Souquere S. Ernoult-Lange M. Chouaib R. (2017). P-body Purification Reveals the Condensation of Repressed mRNA Regulons. Mol. Cell 68, 144–157. 10.1016/j.molcel.2017.09.003 28965817

[B45] Hundley H. A. Bass B. L. (2010). ADAR Editing in Double-Stranded UTRs and Other Noncoding RNA Sequences. Trends Biochem. Sci. 35 (7), 377–383. 10.1016/j.tibs.2010.02.008 20382028 PMC2897959

[B46] Hussain S. P. Hofseth L. J. Harris C. C. (2003). Radical Causes of Cancer. Nat. Rev. Cancer 3, 276–285. 10.1038/nrc1046 12671666

[B47] Hwang Y. E. Baek Y. M. Baek A. Kim D.-E. (2019). Oxidative Stress Causes Alu RNA Accumulation via PIWIL4 Sequestration into Stress Granules. BMB Rep. 52 (3), 196–201. 10.5483/bmbrep.2019.52.3.146 30103846 PMC6476485

[B48] Hyman A. A. Weber C. A. Jülicher F. (2014). Liquid-liquid Phase Separation in Biology. Annu. Rev. Cell Dev. Biol. 30, 39–58. 10.1146/annurev-cellbio-100913-013325 25288112

[B49] Iserman C. Desroches Altamirano C. Jegers C. Friedrich U. Zarin T. Fritsch A. W. (2020). Condensation of Ded1p Promotes a Translational Switch from Housekeeping to Stress Protein Production. Cell 181 (4), 818–831. 10.1016/j.cell.2020.04.009 32359423 PMC7237889

[B50] Ishii T. Hayakawa H. Igawa T. Sekiguchi T. Sekiguchi M. (2018). Specific Binding of PCBP1 to Heavily Oxidized RNA to Induce Cell Death. Proc. Natl. Acad. Sci. USA 115 (26), 6715–6720. 10.1073/pnas.1806912115 29891675 PMC6042155

[B51] Ivanov P. Emara M. M. Villen J. Gygi S. P. Anderson P. (2011). Angiogenin-induced tRNA Fragments Inhibit Translation Initiation. Mol. Cell 43, 613–623. 10.1016/j.molcel.2011.06.022 21855800 PMC3160621

[B52] Ivanov P. Kedersha N. Kedersha P. (2019). Stress Granules and Processing Bodies in Translational Control. Cold Spring Harb Perspect. Biol. 11 (5), a032813. 10.1101/cshperspect.a032813 30082464 PMC6496347

[B53] Jain S. Wheeler J. R. Walters R. W. Agrawal A. Barsic A. Parker R. (2016). ATPase-Modulated Stress Granules Contain a Diverse Proteome and Substructure. Cell 164 (3), 487–498. 10.1016/j.cell.2015.12.038 26777405 PMC4733397

[B54] Jezowska-Bojczuk M. Szczepanik W. Leśniak W. Ciesiołka J. Wrzesiński J. Bal W. (2002). DNA and RNA Damage by Cu(II)-amikacin Complex. Eur. J. Biochem. 269, 5547–5556. 10.1046/j.1432-1033.2002.03260.x 12423353

[B55] Kato M. Han T. W. Xie S. Shi K. Du X. Wu L. C. (2012). Cell-free Formation of RNA Granules: Low Complexity Sequence Domains Form Dynamic Fibers within Hydrogels. Cell 149, 753–767. 10.1016/j.cell.2012.04.017 22579281 PMC6347373

[B56] Kedersha N. Anderson P. (2007). Mammalian Stress Granules and Processing Bodies. Methods Enzymol. 431, 61–81. 10.1016/s0076-6879(07)31005-7 17923231

[B57] Kedersha N. Anderson P. (2002). Stress Granules: Sites of mRNA Triage that Regulate mRNA Stability and Translatability. Biochem. Soc. Trans. 30, 963–969. 10.1042/bst0300963 12440955

[B58] Kedersha N. Chen S. Gilks N. Li W. Miller I. J. Stahl J. (2002). Evidence that Ternary Complex (eIF2-GTP-tRNAi Met)-Deficient Preinitiation Complexes Are Core Constituents of Mammalian Stress Granules. MBoC 13, 195–210. 10.1091/mbc.01-05-0221 11809833 PMC65082

[B59] Kedersha N. Cho M. R. Li W. Yacono P. W. Chen S. Gilks N. (2000). Dynamic Shuttling of TIA-1 Accompanies the Recruitment of mRNA to Mammalian Stress Granules. J. Cell Biol. 151, 1257–1268. 10.1083/jcb.151.6.1257 11121440 PMC2190599

[B60] Kedersha N. L. Gupta M. Li W. Miller I. Anderson P. (1999). RNA-binding Proteins Tia-1 and Tiar Link the Phosphorylation of Eif-2α to the Assembly of Mammalian Stress Granules. J. Cel Biol. 147, 1431–1442. 10.1083/jcb.147.7.1431 PMC2174242 10613902

[B61] Kedersha N. Panas M. D. Achorn C. A. Lyons S. Tisdale S. Hickman T. (2016). G3BP-Caprin1-USP10 Complexes Mediate Stress Granule Condensation and Associate with 40S Subunits. J. Cell Biol. 212, 845–860. 10.1083/jcb.201508028 27022092 PMC4810302

[B62] Kedersha N. Stoecklin G. Ayodele M. Yacono P. Lykke-Andersen J. Fritzler M. J. (2005). Stress Granules and Processing Bodies Are Dynamically Linked Sites of mRNP Remodeling. J. Cell Biol. 169, 871–884. 10.1083/jcb.200502088 15967811 PMC2171635

[B63] Khong A. Matheny T. Jain S. Mitchell S. F. Wheeler J. R. Parker R. (2017). The Stress Granule Transcriptome Reveals Principles of mRNA Accumulation in Stress Granules. Mol. Cel 68, 808–820. 10.1016/j.molcel.2017.10.015 PMC5728175 29129640

[B64] Khong A. Parker R. (2018). mRNP Architecture in Translating and Stress Conditions Reveals an Ordered Pathway of mRNP Compaction. J. Cell Biol. 217 (12), 4124–4140. 10.1083/jcb.201806183 30322972 PMC6279387

[B65] Kiebler M. A. Hemraj I. Verkade P. Köhrmann M. Fortes P. Marión R. M. (1999). The Mammalian Staufen Protein Localizes to the Somatodendritic Domain of Cultured Hippocampal Neurons: Implications for its Involvement in mRNA Transport. J. Neurosci. 19, 288–297. 10.1523/jneurosci.19-01-00288.1999 9870958 PMC6782358

[B66] Kim D. D. Y. Kim T. Walsh T. Kobayashi Y. Matise T. Buyske S. (2004). Widespread RNA Editing of Embedded Alu Elements in the Human Transcriptome. Genome Res. 14, 1719–1725. 10.1101/gr.2855504 15342557 PMC515317

[B67] Kim Y. K. Furic L. Desgroseillers L. Maquat L. E. (2005). Mammalian Staufen1 Recruits Upf1 to Specific mRNA 3′UTRs So as to Elicit mRNA Decay. Cell 120, 195–208. 10.1016/j.cell.2004.11.050 15680326

[B68] Kimball S. R. Horetsky R. L. Ron D. Jefferson L. S. Harding H. P. (2003). Mammalian Stress Granules Represent Sites of Accumulation of Stalled Translation Initiation Complexes. Am. J. Physiol. Cell Physiol. 284, 273–284. 10.1152/ajpcell.00314.2002 12388085

[B69] Knezevic I. Patel A. Sundaresan N. R. Gupta M. P. Solaro R. J. Nagalingam R. S. (2012). A Novel Cardiomyocyte-Enriched MicroRNA, miR-378, Targets Insulin-like Growth Factor 1 Receptor. J. Biol. Chem. 287, 12913–12926. 10.1074/jbc.m111.331751 22367207 PMC3339988

[B70] Kong Q. Lin C.-L. G. (2010). Oxidative Damage to RNA: Mechanisms, Consequences, and Diseases. Cel. Mol. Life Sci. 67 (11), 1817–1829. 10.1007/s00018-010-0277-y PMC3010397 20148281

[B71] Kong Q. Shan X. Chang Y. Tashiro H. Lin C.-L. G. (2008). RNA Oxidation: a Contributing Factor or an Epiphenomenon in the Process of Neurodegeneration. Free Radic. Res. 42, 773–777. 10.1080/10715760802311187 18661427

[B72] Lamers M. M. van den Hoogen B. G. Haagmans B. L. (2019). ADAR1: "Editor-In-Chief" of Cytoplasmic Innate Immunity. Front. Immunol. 10, 1763. 10.3389/fimmu.2019.01763 31404141 PMC6669771

[B73] Laver J. D. Li X. Ancevicius K. Westwood J. T. Smibert C. A. Morris Q. D. (2013). Genome-wide Analysis of Staufen-Associated mRNAs Identifies Secondary Structures that Confer Target Specificity. Nucleic Acids Res. 41, 9438–9460. 10.1093/nar/gkt702 23945942 PMC3814352

[B74] Lee S. B. Kim J. J. Chung J. S. Lee M.-S. Lee K.-H. Kim B. S. (2011). Romo1 Is a Negative-Feedback Regulator of Myc. J. Cell Sci. 124, 1911–1924. 10.1242/jcs.079996 21558421

[B75] Li P. Banjade S. Cheng H.-C. Kim S. Chen B. Guo L. (2012). Phase Transitions in the Assembly of Multivalent Signalling Proteins. Nature 483, 336–340. 10.1038/nature10879 22398450 PMC3343696

[B76] Li Y. R. King O. D. Shorter J. Gitler A. D. (2013). Stress Granules as Crucibles of ALS Pathogenesis. J. Cel Biol 201 (3), 361–372. 10.1083/jcb.201302044 PMC3639398 23629963

[B77] Li Z. Chen X. Liu Z. Ye W. Li L. Qian L. (2020). Recent Advances: Molecular Mechanism of RNA Oxidation and its Role in Various Diseases. Front. Mol. Biosci. 7, 184. 10.3389/fmolb.2020.00184 32850971 PMC7413073

[B78] Li Z. Wu J. Deleo C. (2006). RNA Damage and Surveillance under Oxidative Stress. IUBMB Life (International Union Biochem. Mol. Biol. Life) 58, 581–588. 10.1080/15216540600946456 17050375

[B79] Liddicoat B. J. Piskol R. Chalk A. M. Ramaswami G. Higuchi M. Hartner J. C. (2015). RNA Editing by ADAR1 Prevents Mda5 Sensing of Endogenous Dsrna as Nonself. Science 349, 1115–1120. 10.1126/science.aac7049 26275108 PMC5444807

[B80] Liu M. (2012). RNA Oxidative Damage and Ribosomal RNA Surveillance under Oxidative Stress. [Ph.D dissertation]. Boca Raton, FL: Florida Atlantic University. Publication # 3355620. 10.1158/1538-7445.am2012-2223

[B81] Liu M. Alluri R. K. Fu H. Li Z. (2020). “RNA Oxidative Damage Is Affected by RNA Structures [Conference Presentation],” in The 25th Annual Meeting of the RNA Society, May 26–31 (RNA Society, 2020), Online.

[B82] Liu M. Gong X. Alluri R. K. Wu J. Sablo T. Li Z. (2012). Characterization of RNA Damage under Oxidative Stress in *Escherichia coli* . Biol. Chem. 393, 123–132. 10.1515/hsz-2011-0247 22718628 PMC3404489

[B83] Lou Q. Hu Y. Ma Y. Dong Z. (2019). RNA Interference May Suppress Stress Granule Formation by Preventing Argonaute 2 Recruitment. Am. J. Physiol.-Cell Physiol. 316 (1), C81–C91. 10.1152/ajpcell.00251.2018 30404558 PMC6383145

[B84] Loughlin F. E. West D. L. Gunzburg M. J. Waris S. Crawford S. A. Wilce M. C. J. (2021). Tandem RNA Binding Sites Induce Self-Association of the Stress Granule Marker Protein TIA-1. Nucleic Acids Res. 49 (5), 2403–2417. 10.1093/nar/gkab080 33621982 PMC7969032

[B85] Lushchak V. I. (2014). Free Radicals, Reactive Oxygen Species, Oxidative Stress and its Classification. Chem.-Biol. Interact. 224, 164–175. 10.1016/j.cbi.2014.10.016 25452175

[B86] Mahboubi H. Stochaj U. (2017). Cytoplasmic Stress Granules: Dynamic Modulators of Cell Signaling and Disease. Biochim. Biophys. Acta (Bba) - Mol. Basis Dis. 1863, 884–895. 10.1016/j.bbadis.2016.12.022 28095315

[B87] Mannion N. M. Greenwood S. M. Young R. Cox S. Brindle J. Read D. (2014). The RNA-Editing Enzyme ADAR1 Controls Innate Immune Responses to RNA. Cell Rep. 9 (4), 1482–1494. 10.1016/j.celrep.2014.10.041 25456137 PMC4542304

[B88] Markmiller S. Soltanieh S. Server K. L. Mak R. Jin W. Fang M. Y. (2018). Context-Dependent and Disease-specific Diversity in Protein Interactions within Stress Granules. Cell 172 (3), 590–604. 10.1016/j.cell.2017.12.032 29373831 PMC5969999

[B89] Martindale J. L. Holbrook N. J. (2002). Cellular Response to Oxidative Stress: Signaling for Suicide and Survival. J. Cell. Physiol. 192, 1–15. 10.1002/jcp.10119 12115731

[B90] Martinet W. de Meyer G. R. Y. Herman A. G. Kockx M. M. (2004). Reactive Oxygen Species Induce RNA Damage in Human Atherosclerosis. Eur. J. Clin. Invest. 34, 323–327. 10.1111/j.1365-2362.2004.01343.x 15147328

[B91] Mateju D. Eichenberger B. Voigt F. Eglinger J. Roth G. Chao J. A. (2020). Single-Molecule Imaging Reveals Translation of mRNAs Localized to Stress Granules. Cell 183 (7), 1801–1812. 10.1016/j.cell.2020.11.010 33308477

[B92] Matheny T. Rao B. S. Parker R. (2019). Transcriptome-wide Comparison of Stress Granules and P-Bodies Reveals that Translation Plays a Major Role in RNA Partitioning. Mol. Cel Biol 39, 00313–00319. 10.1128/mcb.00313-19 PMC6879202 31591142

[B93] McCormick C. Khaperskyy D. A. (2017). Translation Inhibition and Stress Granules in the Antiviral Immune Response. Nat. Rev. Immunol. 17, 647–660. 10.1038/nri.2017.63 28669985

[B94] McKinlay A. Gerard W. Fields S. (2012). Global Analysis of RNA Oxidation in *Saccharomyces cerevisiae* . Biotechniques 52, 109–111. 10.2144/000113801 22313409 PMC3339438

[B95] Micklem D. R. Adams J. Grunert S. St. Johnston D. (2000). Distinct Roles of Two Conserved Staufen Domains in Oskar mRNA Localization and Translation. EMBO J. 19, 1366–1377. 10.1093/emboj/19.6.1366 10716936 PMC305677

[B96] Mollet S. Cougot N. Wilczynska A. Dautry F. Kress M. Bertrand E. (2008). Translationally Repressed mRNA Transiently Cycles through Stress Granules during Stress. MBoC 19, 4469–4479. 10.1091/mbc.e08-05-0499 18632980 PMC2555929

[B97] Molliex A. Temirov J. Lee J. Coughlin M. Kanagaraj A. P. Kim H. J. (2015). Phase Separation by Low Complexity Domains Promotes Stress Granule Assembly and Drives Pathological Fibrillization. Cell 163 (1), 123–133. 10.1016/j.cell.2015.09.015 26406374 PMC5149108

[B98] Namkoong S. Ho A. Woo Y. M. Kwak H. Lee J. H. (2018). Systematic Characterization of Stress-Induced RNA Granulation. Mol. Cell 70, 175–187. 10.1016/j.molcel.2018.02.025 29576526 PMC6359928

[B99] Nevo-Caspi Y. Amariglio N. Rechavi G. Paret G. (2011). A-to-I RNA Editing Is Induced upon Hypoxia. Shock 35 (6), 585–589. 10.1097/shk.0b013e31820fe4b7 21330951

[B100] Ng S. K. Weissbach R. Ronson G. E. Scadden A. D. J. (2013). Proteins that Contain a Functional Z-DNA-Binding Domain Localize to Cytoplasmic Stress Granules. Nucleic Acids Res. 41 (21), 9786–9799. 10.1093/nar/gkt750 23982513 PMC3834823

[B161] Nover L. Scharf K. D. Neumann D. (1989). Cytoplasmic Heat Shock Granules are Formed from Precursor Particles and are Associated with a Specific Set of mRNAs. Mol. Cell Biol. 9, 1298–1308. 10.1128/mcb.9.3.1298 2725500 PMC362722

[B101] Nunomura A. Perry G. (2020). RNA and Oxidative Stress in Alzheimer's Disease: Focus on microRNAs. Oxid Med. Cel Longev 2020, 2638130. 10.1155/2020/2638130 PMC7721489 33312335

[B102] Nunomura A. Perry G. Aliev G. Hirai K. Takeda A. Balraj E. K. (2001). Oxidative Damage Is the Earliest Event in Alzheimer Disease. J. Neuropathol. Exp. Neurol. 60, 759–767. 10.1093/jnen/60.8.759 11487050

[B103] Okonski K. M. Samuel C. E. (2013). Stress Granule Formation Induced by Measles Virus Is Protein Kinase PKR Dependent and Impaired by RNA Adenosine Deaminase ADAR1. J. Virol. 87, 756–766. 10.1128/jvi.02270-12 23115276 PMC3554044

[B104] Onomoto K. Yoneyama M. Fung G. Kato H. Fujita T. (2014). Antiviral Innate Immunity and Stress Granule Responses. Trends Immunol. 35 (9), 420–428. 10.1016/j.it.2014.07.006 25153707 PMC7185371

[B105] Orrù S. Coni P. Floris A. Littera R. Carcassi C. Sogos V. (2016). Reduced Stress Granule Formation and Cell Death in Fibroblasts with the A382T Mutation of TARDBP Gene: Evidence for Loss of TDP-43 Nuclear Function. Hum. Mol. Genet. 25 (20), 4473–4483. 10.1093/hmg/ddw276 28172957

[B106] Park E. Maquat L. E. (2013). Staufen-mediated mRNA Decay. WIREs RNA 4, 423–435. 10.1002/wrna.1168 23681777 PMC3711692

[B107] Park Y.-J. Choi D. W. Cho S. W. Han J. Yang S. Choi C. Y. (2020). Stress Granule Formation Attenuates RACK1-Mediated Apoptotic Cell Death Induced by Morusin. Ijms 21 (15), 5360. 10.3390/ijms21155360 PMC7432505 32731602

[B108] Patel A. Lee H. O. Jawerth L. Maharana S. Jahnel M. Hein M. Y. (2015). A Liquid-To-Solid Phase Transition of the ALS Protein FUS Accelerated by Disease Mutation. Cell 162, 1066–1077. 10.1016/j.cell.2015.07.047 26317470

[B109] Poblete-Durán N. Prades-Pérez Y. Vera-Otarola J. Soto-Rifo R. Valiente-Echeverría F. (2016). Who Regulates Whom? an Overview of RNA Granules and Viral Infections. Viruses 8 (7), 180. 10.3390/v8070180 PMC4974515 27367717

[B110] Poli G. Leonarduzzi G. Biasi F. Chiarpotto E. (2004). Oxidative Stress and Cell Signalling. Curr. Med. Chem. 11 (9), 1163–1182. 10.2174/0929867043365323 15134513

[B111] Poulsen H. Nilsson J. Damgaard C. K. Egebjerg J. Kjems J. (2001). CRM1 Mediates the export of ADAR1 through a Nuclear export Signal within the Z-DNA Binding Domain. Mol. Cell. Biol. 21, 7862–7871. 10.1128/mcb.21.22.7862-7871.2001 11604520 PMC99958

[B112] Prasanth K. V. Prasanth S. G. Xuan Z. Hearn S. Freier S. M. Bennett C. F. (2005). Regulating Gene Expression through RNA Nuclear Retention. Cell 123, 249–263. 10.1016/j.cell.2005.08.033 16239143

[B113] Protter D. S. W. Parker R. (2016). Principles and Properties of Stress Granules. Trends Cell Biology 26 (9), 668–679. 10.1016/j.tcb.2016.05.004 PMC4993645 27289443

[B114] Ramaswami M. Taylor J. P. Parker R. (2013). Altered Ribostasis: RNA-Protein Granules in Degenerative Disorders. Cell 154 (4), 727–736. 10.1016/j.cell.2013.07.038 23953108 PMC3811119

[B115] Roden C. Gladfelter A. S. (2021). RNA Contributions to the Form and Function of Biomolecular Condensates. Nat. Rev. Mol. Cell Biol. 22 (3), 183–195. 10.1038/s41580-020-0264-6 32632317 PMC7785677

[B116] Ryter S. W. Kim H. P. Hoetzel A. Park J. W. Nakahira K. Wang X. (2007). Mechanisms of Cell Death in Oxidative Stress. Antioxid. Redox Signaling 9 (1), 49–89. 10.1089/ars.2007.9.49 17115887

[B117] Sayre L. Smith M. Perry G. (2001). Chemistry and Biochemistry of Oxidative Stress in Neurodegenerative Disease. Cmc 8, 721–738. 10.2174/0929867013372922 11375746

[B118] Scadden A. D. J. (2007). Inosine-containing dsRNA Binds a Stress-granule-like Complex and Downregulates Gene Expression in Trans. Mol. Cell 28, 491–500. 10.1016/j.molcel.2007.09.005 17996712 PMC2075533

[B119] Scadden A. D. J. (2005). The RISC Subunit Tudor-SN Binds to Hyper-Edited Double-Stranded RNA and Promotes its Cleavage. Nat. Struct. Mol. Biol. 12, 489–496. 10.1038/nsmb936 15895094

[B120] Sen N. D. Zhou F. Ingolia N. T. Hinnebusch A. G. (2015). Genome-wide Analysis of Translational Efficiency Reveals Distinct but Overlapping Functions of Yeast DEAD-Box RNA Helicases Ded1 and eIF4A. Genome Res. 25 (8), 1196–1205. 10.1101/gr.191601.115 26122911 PMC4510003

[B121] Shan X. Chang Y. Glenn Lin C. l. (2007). Messenger RNA Oxidation Is an Early Event Preceding Cell Death and Causes Reduced Protein Expression. FASEB j. 21, 2753–2764. 10.1096/fj.07-8200com 17496160

[B122] Shan X. Tashiro H. Lin C.-l. G. (2003). The Identification and Characterization of Oxidized RNAs in Alzheimer's Disease. J. Neurosci. 23, 4913–4921. 10.1523/jneurosci.23-12-04913.2003 12832513 PMC6741200

[B123] Sheth U. Parker R. (2003). Decapping and Decay of Messenger RNA Occur in Cytoplasmic Processing Bodies. Science 300, 805–808. 10.1126/science.1082320 12730603 PMC1876714

[B124] Sheth U. Parker R. (2006). Targeting of Aberrant mRNAs to Cytoplasmic Processing Bodies. Cell 125, 1095–1109. 10.1016/j.cell.2006.04.037 16777600 PMC1858659

[B125] Si W. Ye S. Ren Z. Liu X. Wu Z. Li Y. (2019). miR-335 Promotes Stress Granule Formation to Inhibit Apoptosis by Targeting ROCK2 in Acute Ischemic Stroke. Int. J. Mol. Med. 43 (3), 1452–1466. 10.3892/ijmm.2019.4073 30747210 PMC6365079

[B126] Singh S. Dwarakanath B. Mathew T. (2004). DNA Ligand Hoechst-33342 Enhances UV Induced Cytotoxicity in Human Glioma Cell Lines. J. Photochem. Photobiol. B: Biol. 77, 45–54. 10.1016/s1011-1344(04)00122-8 15542361

[B127] Singh U. Jialal I. (2006). Oxidative Stress and Atherosclerosis. Pathophysiology 13 (3), 129–142. 10.1016/j.pathophys.2006.05.002 16757157

[B128] Somasekharan S. P. El-Naggar A. Leprivier G. Cheng H. Hajee S. Grunewald T. G. P. (2015). YB-1 Regulates Stress Granule Formation and Tumor Progression by Translationally Activating G3BP1. J. Cell Biol 208, 913–929. 10.1083/jcb.201411047 25800057 PMC4384734

[B129] Souquere S. Mollet S. Kress M. Dautry F. Pierron G. Weil D. (2009). Unravelling the Ultrastructure of Stress Granules and Associated P-Bodies in Human Cells. J. Cell Sci 122, 3619–3626. 10.1242/jcs.054437 19812307

[B130] Stöhr N. Lederer M. Reinke C. Meyer S. Hatzfeld M. Singer R. H. (2006). ZBP1 Regulates mRNA Stability during Cellular Stress. J. Cell Biol. 175, 527–534. 10.1083/jcb.200608071 17101699 PMC2064588

[B131] Strehblow A. Hallegger M. Jantsch M. F. (2002). Nucleocytoplasmic Distribution of Human RNA-Editing Enzyme ADAR1 Is Modulated by Double-Stranded RNA-Binding Domains, a Leucine-Rich export Signal, and a Putative Dimerization Domain. MBoC 13, 3822–3835. 10.1091/mbc.e02-03-0161 12429827 PMC133595

[B132] Takahashi M. Higuchi M. Matsuki H. Yoshita M. Ohsawa T. Oie M. (2013). Stress Granules Inhibit Apoptosis by Reducing Reactive Oxygen Species Production. Mol. Cell. Biol. 33 (4), 815–829. 10.1128/mcb.00763-12 23230274 PMC3571346

[B133] Tanaka M. Chock P. B. Stadtman E. R. (2007). Oxidized Messenger RNA Induces Translation Errors. Proc. Natl. Acad. Sci. 104, 66–71. 10.1073/pnas.0609737104 17190801 PMC1765478

[B134] Tanaka M. Song H. Küpfer P. A. Leumann C. J. Sonntag W. E. Sonntag W. E. (2011). An Assay for RNA Oxidation Induced Abasic Sites Using the Aldehyde Reactive Probe. Free Radic. Res. 45, 237–247. 10.3109/10715762.2010.535529 21062214 PMC3058411

[B135] Tang S. J. Meulemans D. Vazquez L. Colaco N. Schuman E. (2001). A Role for a Rat Homolog of Staufen in the Transport of RNA to Neuronal Dendrites. Neuron 32, 463–475. 10.1016/s0896-6273(01)00493-7 11709157

[B136] Thomas M. G. Tosar L. J. M. Desbats M. A. Leishman C. C. Boccaccio G. L. (2009). Mammalian Staufen 1 Is Recruited to Stress Granules and Impairs Their Assembly. J. Cell Sci. 122, 563–573. 10.1242/jcs.038208 19193871 PMC2714435

[B137] Thomas M. G. Tosar L. J. M. Loschi M. Pasquini J. M. Correale J. Kindler S. (2005). Staufen Recruitment into Stress Granules Does Not Affect Early mRNA Transport in Oligodendrocytes. MBoC 16, 405–420. 10.1091/mbc.e04-06-0516 15525674 PMC539183

[B138] Thompson D. M. Lu C. Green P. J. Parker R. (2008). tRNA Cleavage Is a Conserved Response to Oxidative Stress in Eukaryotes. RNA 14, 2095–2103. 10.1261/rna.1232808 18719243 PMC2553748

[B139] Thompson D. M. Parker R. (2009). The RNase Rny1p Cleaves tRNAs and Promotes Cell Death during Oxidative Stress in *Saccharomyces cerevisiae* . J. Cell Biol. 185, 43–50. 10.1083/jcb.200811119 19332891 PMC2700514

[B140] Tian S. Curnutte H. A. Trcek T. (2020). RNA Granules: A View from the RNA Perspective. Molecules 25 (14), 3130. 10.3390/molecules25143130 PMC7397151 32650583

[B141] Tourrière H. Chebli K. Zekri L. Courselaud B. Blanchard J. M. Bertrand E. (2003). The RasGAP-Associated Endoribonuclease G3BP Assembles Stress Granules. J. Cel Biol. 160, 823–831. 10.1083/jcb.200212128 PMC2173781 12642610

[B142] Tranter M. Helsley R. N. Paulding W. R. McGuinness M. Brokamp C. Haar L. (2011). Coordinated Post-transcriptional Regulation of Hsp70.3 Gene Expression by MicroRNA and Alternative Polyadenylation*. J. Biol. Chem. 286, 29828–29837. 10.1074/jbc.m111.221796 21757701 PMC3191024

[B143] Valiente-Echeverría F. Melnychuk L. Mouland A. J. (2012). Viral Modulation of Stress Granules. Virus. Res. 169, 430–437. 10.1016/j.virusres.2012.06.004 22705970 PMC7114395

[B144] Van Treeck B. Parker R. (2019). Principles of Stress Granules Revealed by Imaging Approaches. Cold Spring Harb Perspect. Biol. 11 (2), a033068. 10.1101/cshperspect.a033068 30709880 PMC6360856

[B145] Vitali P. Scadden A. D. J. (2010). Double-stranded RNAs Containing Multiple IU Pairs Are Sufficient to Suppress Interferon Induction and Apoptosis. Nat. Struct. Mol. Biol. 17 (9), 1043–1050. 10.1038/nsmb.1864 20694008 PMC2935675

[B146] Vogiatzi G. Tousoulis D. Stefanadis C. (2009). The Role of Oxidative Stress in Atherosclerosis. Hellenic J. Cardiol. 50(5), 402–409. 19767282

[B147] Wang Q. Li X. Qi R. Billiar T. (2017). RNA Editing, ADAR1, and the Innate Immune Response. Genes 8 (1), 41. 10.3390/genes8010041 PMC5295035 28106799

[B148] Weissbach R. Scadden A. D. J. (2012). Tudor-SN and ADAR1 Are Components of Cytoplasmic Stress Granules. RNA 18, 462–471. 10.1261/rna.027656.111 22240577 PMC3285934

[B149] White J. P. Lloyd R. E. (2012). Regulation of Stress Granules in Virus Systems. Trends Microbiol. 20, 175–183. 10.1016/j.tim.2012.02.001 22405519 PMC3322245

[B150] Wippich F. Bodenmiller B. Trajkovska M. G. Wanka S. Aebersold R. Pelkmans L. (2013). Dual Specificity Kinase DYRK3 Couples Stress Granule Condensation/dissolution to mTORC1 Signaling. Cell 152, 791–805. 10.1016/j.cell.2013.01.033 23415227

[B151] Wolozin B. Ivanov P. (2019). Stress Granules and Neurodegeneration. Nat. Rev. Neurosci. 20, 649–666. 10.1038/s41583-019-0222-5 31582840 PMC6986315

[B152] Wu J. Li Z. (2008). Human Polynucleotide Phosphorylase Reduces Oxidative RNA Damage and Protects HeLa Cell against Oxidative Stress. Biochem. Biophysical Res. Commun. 372, 288–292. 10.1016/j.bbrc.2008.05.058 PMC2531134 18501193

[B153] Yamasaki S. Ivanov P. Hu G.-f. Anderson P. (2009). Angiogenin Cleaves tRNA and Promotes Stress-Induced Translational Repression. J. Cell Biol 185, 35–42. 10.1083/jcb.200811106 19332886 PMC2700517

[B154] Yang J. Välineva T. Hong J. Bu T. Yao Z. Jensen O. N. (2007). Transcriptional Co-activator Protein P100 Interacts with snRNP Proteins and Facilitates the Assembly of the Spliceosome. Nucleic Acids Res. 35 (13), 4485–4494. 10.1093/nar/gkm470 17576664 PMC1935017

[B155] Yang S. Deng P. Zhu Z. Zhu J. Wang G. Zhang L. (2014). Adenosine Deaminase Acting on RNA 1 Limits Rig-I RNA Detection and Suppresses Ifn Production Responding to Viral and Endogenous RNAs. J.I. 193, 3436–3445. 10.4049/jimmunol.1401136 PMC4169998 25172485

[B156] Yoneyama M. Jogi M. Onomoto K. (2016). Regulation of Antiviral Innate Immune Signaling by Stress-Induced RNA Granules. J. Biochem. 159 (3), 279–286. 10.1093/jb/mvv122 26748340 PMC4763080

[B157] Yu Z. Chen T. Cao X. (2015). RNA Editing by ADAR1 marks dsRNA as "self". Cell. Res. 25, 1283–1284. 10.1038/cr.2015.135 26596786 PMC4670992

[B158] Zhan Y. Dhaliwal J. S. Adjibade P. Uniacke J. Mazroui R. Zerges W. (2015). Localized Control of Oxidized RNA. J. Cell Sci 128, 4210–4219. 10.1242/jcs.175232 26449969

[B159] Zhang J. Perry G. Smith M. A. Robertson D. Olson S. J. Graham D. G. (1999). Parkinson's Disease Is Associated with Oxidative Damage to Cytoplasmic DNA and RNA in Substantia Nigra Neurons. Am. J. Pathol. 154, 1423–1429. 10.1016/s0002-9440(10)65396-5 10329595 PMC1866598

[B160] Zheng D. Ezzeddine N. Chen C.-Y. A. Zhu W. He X. Shyu A.-B. (2008). Deadenylation Is Prerequisite for P-Body Formation and mRNA Decay in Mammalian Cells. J. Cell Biol 182, 89–101. 10.1083/jcb.200801196 18625844 PMC2447901

